# Silibinin Attenuates Experimental Periodontitis by Downregulation of Inflammation and Oxidative Stress

**DOI:** 10.1155/2023/5617800

**Published:** 2023-02-15

**Authors:** Xumin Li, Runqi Zhou, Yue Han, Jun Zeng, Lixi Shi, Yixin Mao, Xiaoyu Sun, Yinghui Ji, Xiaorong Zhang, Yang Chen, Richard T. Jaspers, Gang Wu, Shengbin Huang, Tim Forouzanfar

**Affiliations:** ^1^Department of Prosthodontics, School and Hospital of Stomatology, Wenzhou Medical University, Wenzhou 325000, China; ^2^Institute of Stomatology, School and Hospital of Stomatology, Wenzhou Medical University, Wenzhou 325000, China; ^3^Department of Oral and Maxillofacial Surgery/Pathology, Amsterdam UMC and Academic Centre for Dentistry Amsterdam (ACTA), Vrije Universiteit Amsterdam (VUA), Amsterdam Movement Science, De Boelelaan, 1117 Amsterdam, Netherlands; ^4^Laboratory for Myology, Faculty of Behavioral and Movement Sciences, Vrije Universiteit Amsterdam (VUA), Amsterdam Movement Sciences, De Boelelaan 1108, Netherlands; ^5^Shantou Centre Hospital, Shantou, China; ^6^Department of Periodontics, School and Hospital of Stomatology, Wenzhou Medical University, Wenzhou, China; ^7^Department of Stomatology, Dongyang People's Hospital, Jinhua, China; ^8^Department of Endodontics, School and Hospital of Stomatology, Wenzhou Medical University, Wenzhou, China; ^9^Department of Oral Cell Biology, Academic Centre for Dentistry Amsterdam (ACTA), University of Amsterdam (UvA) and Vrije Universiteit Amsterdam (VU), Amsterdam, Netherlands

## Abstract

Periodontitis is an oral microbiota-induced inflammatory disease, in which inflammation and oxidative stress play a critical role. Silibinin (SB), a Silybum marianum-derived compound, exhibits strong anti-inflammatory and antioxidative properties. We adopted a rat ligature-induced periodontitis model and a lipopolysaccharide- (LPS-) stimulated human periodontal ligament cells (hPDLCs) model to evaluate the protective effects of SB. In the *in vivo* model, SB reduced alveolar bone loss and apoptosis of PDLCs in the periodontal tissue. SB also maintained the expression of nuclear factor-E2-related factor 2 (Nrf2), a key regulator of cellular resistance to oxidative stress, and attenuated lipid, protein, and DNA oxidative damages in the periodontal lesion area. Meanwhile, in the *in vitro* model, SB administration reduced the production of intracellular reactive oxidative species (ROS). Furthermore, SB exerted a strong anti-inflammatory property in both *in vivo* and *in vitro* models by inhibiting the expression of inflammatory mediators including nuclear factor-*κ*B (NF-*κ*B) as well as nucleotide binding oligomerization domain- (NOD-) like receptor family pyrin domain-containing 3 (NLRP3) and downregulating the levels of proinflammatory cytokines. This study, for the first time, demonstrates that SB exhibits the anti-inflammatory and antioxidative properties against periodontitis by downregulating the expression of NF-*κ*B and NLRP3 and upregulating Nrf2 expression, suggesting a promising potential clinical application of SB in periodontitis.

## 1. Introduction

Periodontitis is an oral microbiota-induced inflammatory disease and is symbolized by the destruction of periodontal tissues, such as alveolar bone and periodontal ligament (PDL) [1]. Periodontitis is one of the most common oral diseases worldwide and has become the main cause of tooth loss in adults. Furthermore, periodontitis has also been proven to be associated with the onset and progression of many systemic diseases, such as cardiovascular diseases, respiratory diseases, cognitive impairment, diabetes mellitus, and chronic kidney disease [2]. Pathogens for periodontitis, such as *Porphyromonas gingivalis* (*P. gingivalis*) and *Aggregatibacter actinomycetemcomitans*, strongly activate host-mediated inflammatory responses through both invading into the periodontal tissue and producing pathogenic factors, such as lipopolysaccharide (LPS) [3]. The inflammatory cells recruited by host immune response (such as neutrophils in the initial lesion period, macrophages and T cells in the early lesion period, and B cells as well as plasma cells in the later established and advanced lesion period) secrete both proinflammatory cytokines (e.g., tumor necrosis factor-*α* (TNF-*α*), interleukin- (IL-) 1*β*, and IL-6) [4] and release excessive reactive oxidative species (ROS) [5]. The PDL cells (PDLCs), a group of mesenchymal fibroblast-like cells in PDL tissue for forming, maintaining, and regenerating periodontal tissues [6], can also in response to LPS to produce proinflammatory cytokines [7] and get cellular oxidative damages from LPS [8]. The thereby-triggered inflammation and oxidative stress (OS) further cause the dysfunction and apoptosis of PDLCs [9, 10]. These pathological events eventually lead to periodontal tissue damages, e.g., alveolar bone loss and PDL degeneration [11, 12]. Therefore, it is conceivable that administrating anti-inflammatory and antioxidative agents as an adjuvant therapy may effectively attenuate periodontal tissue damages in periodontitis.

One of such promising agents is silibinin (SB), a natural polyphenolic flavonoid and the major active substance of silymarin [13]—an official medicine protects hepatic functions [14]. SB has been shown to be highly anti-inflammatory and antioxidative [15]. For example, SB may significantly inhibit the expression of proinflammatory cytokines (e.g., TNF-*α* and IL-1*β*) through blocking the activation of signal transducer nuclear factor-*κ*B (NF-*κ*B) and nucleotide binding oligomerization domain- (NOD-) like receptor family pyrin domain-containing 3 (NLRP3) inflammasome [16]. Furthermore, SB can also directly scavenge free radicals and elevate the levels of intracellular antioxidative enzyme [13]. However, the biological effects of SB on the progress of periodontitis remain to be elucidated.

In the present study, we adopted a rat ligature-induced periodontitis model and an LPS-stimulated human PDLC (hPDLC) model to reveal whether SB can attenuate inflammatory and oxidative reactions in periodontitis so as to prevent periodontal destructions.

## 2. Materials and Methods

### 2.1. Animals

Male Wistar rats from the Animal Center of Wenzhou Medical University, weighing 200–270 g, 6–8 weeks old, were used. The rats were acclimatized in temperature-controlled room at 22 ± 2°C, under 12 h light-dark cycles, with free access to water and food for 1 week before experiment started. All experimental protocols were approved by the Animal Ethics Committee of Wenzhou Medical University (WYKQ2015001).

### 2.2. Animal Experimental Design

15 rats were divided randomly into 3 groups of 5 each. The study groups were as follows: C: no treatment, P: ligature, and P+SB: ligature+SB (150 mg kg^−1^day^−1^) administration. For experimental periodontitis model establishment, sterile, 3–0 black braided nylon thread (Surgilon; USS/DG, Norwalk, CT, USA) was placed around the bilateral lower first molars of rats in the P and P+SB groups for two weeks [17]. SB, 150 mg per kilogram body weight per day (according to the previous study [18, 19] and our preliminary experiment), was pretreated 2 weeks before the induction of periodontitis by oral gavage and continued 1 month till the end of 2 weeks' experimental periodontitis.

### 2.3. Stereomicroscopic Examination

Two weeks after ligation, the rats were sacrificed, and the left side of mandible was extracted for stereomicroscopic examination. The distance from the amelocemental junction (ACJ) to the alveolar crest (AC) was measured from the stereomicroscopic images as previously described [17]. We recorded the distance of ACJ-AC in the long axis of both buccal and lingual root surfaces of the first molars. The mean of the recordings for each tooth (expressed in *μ*m) was used as a measure of alveolar bone loss.

### 2.4. Histopathological Evaluation

The right side of the mandible was fixed, decalcified, dehydrated, and embedded in paraffin as previously described [20]. Sections (4 *μ*m thick) were prepared in the mesiodistal plane and used for histopathologic and immunohistochemical assays. Hematoxylin and eosin (H&E) staining was performed for morphologic assessment. Alveolar bone loss in H&E staining images was also measured by the comparison of ACJ-AC distance between groups.

To assess the inflammatory state in the different groups, the number of infiltrated inflammatory cells was counted in 3 random areas (100 × 100 *μ*m) near gingival sulcus, under ×400 magnification [21]. PDLCs and squamous epithelial cells in the field of vision were not counted. In each group, the number of inflammatory cells was analyzed in 5 animals.

### 2.5. Apoptosis Assay

The apoptosis rate of PDLCs in periodontal sections was measured by terminal deoxynucleotidyl transferase dUTP nick end labeling (TUNEL) staining (Roche, Germany) following the manufacturer's instruction. In short, after permeabilization with 0.1% Triton X-100, sections were incubated with TUNEL reaction mixture at 37°C 1 h in the dark. After washing in phosphate-buffered solution (PBS), sections were further incubated with horse-radish peroxidase- (HRP-) conjugated anti-fluorescein antibody for 30 min. Positive cells were labeled in deep brown by the application of diaminobenzidine (DAB) substrate. Then, the sections were counterstained with hematoxylin and mounted. The percentage of apoptotic cells was estimated by the TUNEL-positive cell counts in total cells from three random areas (50 × 50 *μ*m) in PDL at ×400 magnification.

### 2.6. Immunohistochemistry (IHC)

For IHC, sections were stained with anti-4-hydroxy-2-nonenal (4-HNE) (1 : 400), anti-3-nitrotyrosine (3-NT) (1 : 400), and anti-8-hydroxy-deoxyguanosine (8-OHdG) (1 : 200) from Abcam Biotechnology, anti-NF-*κ*B (1 : 400) from Cell Signaling Technology, anti-NLRP3 (1 : 100) from Novus Biologicals, and anti-nuclear factor-E2-related factor 2 (Nrf2) (1 : 400) from Santa Cruz Biotechnology. Detailed immunohistochemistry staining was performed as previously described [20]. In brief, the sections were blocked in the block solution of IHC kit (ZSGB-BIO, China) 15 min and then incubated in the primary antibody at 4°C overnight. The sections were subsequently incubated with a biotinylated goat anti-mouse/rabbit IgG polymer (ZSGB-BIO, China) for 15 min as well as HRP-labeled streptavidin working solution (ZSGB-BIO, China) for 15 min and DAB (ZSGB-BIO, China) as the substrate for 2 min. Counterstaining was performed with hematoxylin. Integrated optical density (IOD) in PDL of each slice were measured at ×400 magnification. The intensity of each protein was measured by the mean optical density (MOD) which is the value of IOD divided by area.

### 2.7. Quantitative Real-Time Polymerase Chain Reaction (qPCR) Analysis of Periodontal Tissue

To analyze messenger RNA (mRNA) expression of a particular set of genes, total RNA was extracted from the gingiva tissue around the ligation region using the TRIzol reagent (Invitrogen, Carlsbad, CA, USA). cDNA was synthesized from 1 mg RNA using PrimeScript RT reagent Kit with gDNA Eraser (Takara, Japan) and quantified by measuring the absorbance at 260 and 280 nm. cDNA amplifications were performed for 30 cycles of 1 minute each at 94°C (denaturation), 60°C (annealing), and 72°C (elongation), and final extension was performed at 72°C for 10 minutes. The sequences of specific primers are listed in Table 1.

### 2.8. Primary Human PDLC (hPDLC) Culture and Identification

The study protocol was approved by the Committee of Research on Human Subjects of the School and Hospital of Stomatology, Wenzhou Medical University (2018001). hPDLCs were obtained from healthy third molars of 3 healthy men with informed consent. PDL tissues from the tooth root were sliced into 1–2 mm^3^ pieces. These little pieces were digested with type I collagenase (Sigma, USA) for 30 min at 37°C and then cultured in *α*-minimum essential medium (*α*-MEM, Gibco, USA) containing 10% fetal bovine serum (FBS, Millipore, USA), 2 mM L-glutamine, 100 U/ml of penicillin, and 100 mg/ml of streptomycin (Gibco, USA) at 37°C in a humidified atmosphere with 5% CO_2_. Cells were passaged when 70%–80% confluence was reached and used at passages 4–8. hPDLCs were identified through immunohistochemical staining for vimentin and cytokeratin (1 : 200, mouse, vimentin, BM0135, cytokeratin, BM0030, BosterBio, Wuhan, China) [6].

### 2.9. Cell Viability Assay of hPDLCs

hPDLCs were cultured in 96-well plates (5 × 10^3^ cells per well) and supplemented with SB (Sigma, USA) concentration from 0 *μ*M to 100 *μ*M for 24 h according to the experimental protocol. After treatment, the viability of hPDLCs was measured by 3(4,5-dimethylthiazol-2-yl)-2,5-diphenyltetrazolium (MTT, Sigma, USA) assay as previously described [22]. Briefly, after treatment, cells were incubated in 100 *μ*l/well serum-free medium supplemented with 10 *μ*l MTT solution (5 mg/ml) at 37°C for 4 h. The formed formazan crystals were dissolved by incubation with 150 *μ*l/well DMSO for 10 min. The absorbance of each well at 570 nm was measured in a microplate reader.

### 2.10. qPCR Analysis of hPDLCs

hPDLCs were treated with 1 *μ*g/ml lipopolysaccharide (LPS) produced from P. gingivalis (Sigma, USA) for 24 h [23] with or without SB (50 *μ*M, 24 h) preincubation. After treatment, qPCR was used to investigate certain gene expression. The sequences of specific primers are listed in Table 2.

### 2.11. Western Blotting Analysis of hPDLCs

After the indicated treatment, hPDLCs were harvested and lysed to extract total protein. Western blotting was performed as previously described [24]. Concentration of primary antibodies are as follows: anti-NF-*κ*B (1 : 1000, Cell Signaling, USA), anti-NLRP3 (1 : 1000, Novus Biologicals, USA), and anti-GAPDH (1 : 1000, Cell Signaling, USA). The following used secondary antibody were horseradish peroxidase-conjugated anti-rabbit IgG antibody (1 : 4000, Invitrogen, USA). The immunoreactive band intensities were quantified using ImageJ software and normalized by GAPDH levels.

### 2.12. ROS Assay of hPDLCs

hPDLCs were seeded on coverslips in 48-well plates (1.5 × 10^4^ cells per well) and treated as indicated. After treatment, 2′,7′-dichlorofluorescin diacetate (DCFH-DA, Thermo Fisher Scientific, USA) were used to assess intracellular ROS generation as previously described [24]. Briefly, hPDLCs were incubated with 10 *μ*M DCFH-DA for 30 min at 37°C and fixed in 4% PFA for 30 min at room temperature. Cells were costained with 4′,6-diamidino-2-phenylindole (DAPI, Sigma, USA) for nuclear localization. Then, fluorescence intensity of cells was assessed by a fluorescence microscope and quantified by ImageJ software.

### 2.13. Statistical Analyses

Data are symmetrically distributed with no skew and expressed as mean ± standard deviation (SD). Statistics were analyzed using GraphPad Prism Software. One-way analysis of variance (ANOVA) was carried out, and Tukey's multiple comparison analysis was used for multiple comparisons. *P* < 0.05 was considered to be significant statistically.

## 3. Results

### 3.1. Body Weight and Fasting Blood Glucose

**Figure 1:**
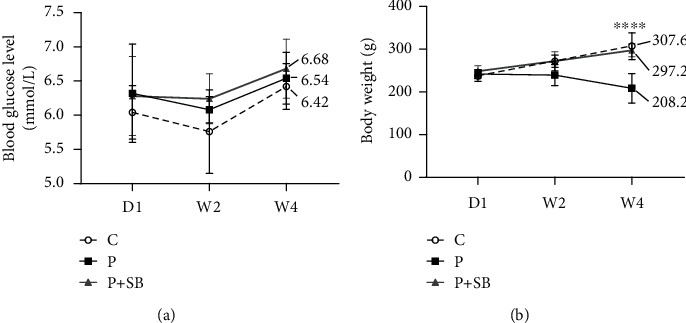
Effects of SB on the (a) body weight (g) and (b) fasting glucose levels (mmol/l) of periodontitis rat model. C: control group; P: experimental periodontitis group; P+SB: SB-administrated experimental periodontitis group; D: day; W: week (*n* = 5). The mean values of W4 in (a) and (b) were marked besides the indicated groups. Data are shown as mean ± SD. ^∗∗∗∗^*P* < 0.0001.

Within a 4-week monitoring time span after the establishment of periodontitis, the fasting blood glucose levels in the rats fluctuated between 5.0 and 7.2 mmol/l (Figure 1(a)) and no significant difference was found among three groups. At week 4, the body weight of the rats in group P was significantly decreased by 32.3% in comparison to those of rats in the control group (*P* < 0.0001). In contrast, the administration of SB to the rats with periodontitis prevented such a decrease (Figure 1(b)).

### 3.2. SB Prevented Periodontal Destructions

**Figure 2:**
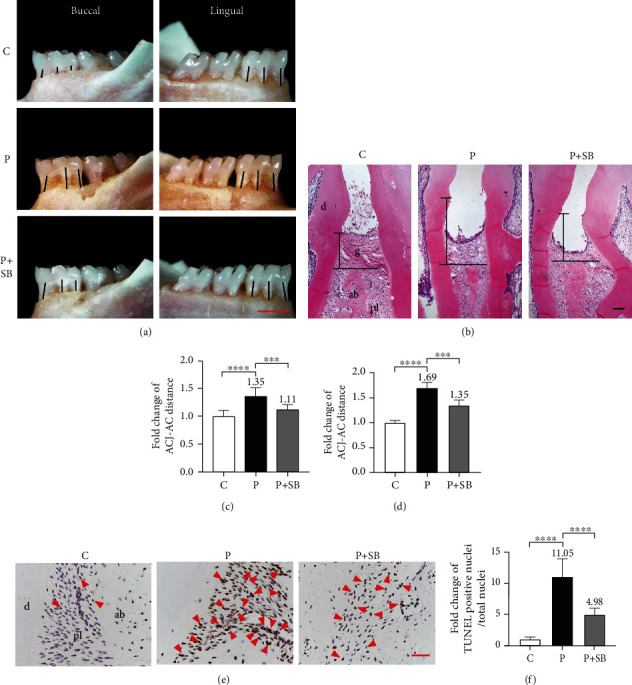
SB prevented periodontal destructions. (a) Macroscopic aspect of mandibles in group C, group P, and group P+SB. The black lines show the distance between amelocemental junction (ACJ) and alveolar crest (AC), scale bar = 1 mm. (b) Hematoxylin and eosin staining images of periodontal tissue from indicated groups. g: gingiva; pl: periodontal ligament; d: dentin; ab: alveolar bone. The black lines indicate the distance between ACJ and AC, scale bar = 100 *μ*m. (c) Quantitative analysis of ACJ-AC distance in each group (*n* = 5). (d) ACJ-AC distance was quantitatively analyzed (*n* = 5). (e) Apoptosis of periodontal ligament cells determined by TUNEL staining. TUNEL-positive nuclei was brown colored and indicated by red solid triangles, and all the nuclei were costained with hematoxylin, scale bar = 50 *μ*m. (f) Quantitative analysis of apoptotic cell numbers (*n* = 5). The mean value of each group but group C was marked on the top of its column. Data are shown as mean ± SD. ^∗∗∗^*P* < 0.001, ^∗∗∗∗^*P* < 0.0001.

We adopted the fold changes of ACJ-AC distance as a parameter to evaluate the alveolar bone loss in the different groups. Rats in group P showed remarkable alveolar bone loss with about 35% increase in comparison with that in group C (*P* < 0.0001), indicating a more severe destruction of periodontal hard tissue in group P. The administration of SB prevented such an increase and maintained the ACJ-AC distance similar as that of group C (Figures 2(a) and 2(c)). Typical H&E-staining of periodontal tissues in group C showed a healthy periodontal structure, in which the alveolar bone ridge and an overlying gingiva containing regularly aligned collagen fibers with its upper edge at ACJ level were detected. In the P group, the gingiva with regularly aligned collagen fibers was replaced by a layer of loose connective tissue with its upper edge significantly shifted downwards (Figure 2(b)). The ACJ-AC in the P group was about 1.7 times that of the C group (*P* < 0.0001) (Figures 2(b) and 2(d)). In contrast, the administration of SB was associated with a limited increase (about 1.3 times) of ACJ-AC distance in comparison with the C group (*P* < 0.001) (Figures 2(b) and 2(d)). We further adopted TUNEL staining to detect the apoptosis of PDLCs as a parameter for the destruction of periodontal soft tissue (Figure 2(e)). In comparison with group C, much more PDLCs in the P group were positive for TUNEL staining, which was attenuated by the administration of SB. The percentage of TUNEL-positive cells in the P group was about 11.1 times that of C group, while the percentage of TUNEL-positive cells in the P+SB group was only 5.0 times that of C group (Figure 2(f)).

### 3.3. SB Attenuated Periodontal Inflammation

**Figure 3:**
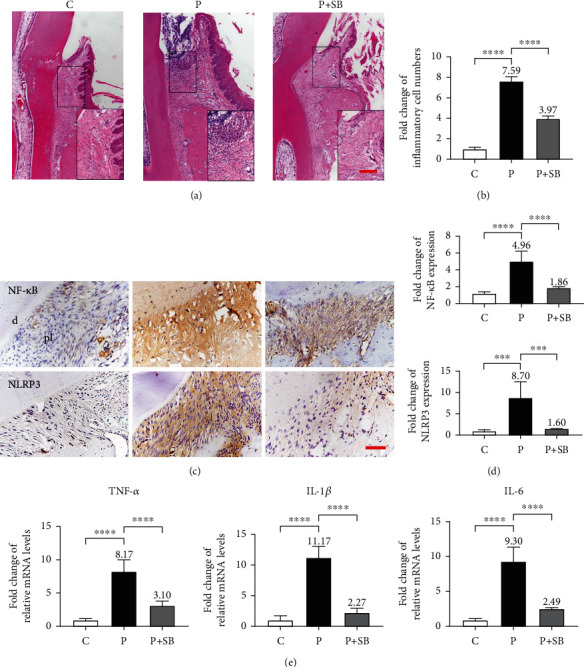
SB attenuated periodontal inflammation. (a) Images of hematoxylin and eosin staining, present the infiltrated inflammatory cells. The lower right images present the black rectangle areas under ×400 magnification, scale bar = 50 *μ*m. (b) The number of infiltrated inflammatory cells was quantitatively analyzed (*n* = 5). (c) Immunohistochemistry staining images of NF-*κ*B and NLRP3. pl: periodontal ligament; d: dentin. Scale bar = 50 *μ*m. (d) Protein expression intensity quantification analysis of NF-*κ*B and NLRP3 (*n* = 5). (e) qPCR assay for the mRNA expression of TNF-*α*, IL-1*β*, and IL-6 in periodontal soft tissues. GAPDH was used as a housekeeping gene. The mean value of each group but group C was marked on the top of its column. Data are shown as mean ± SD. ^∗∗∗^*P* < 0.001, ^∗∗∗∗^*P* < 0.0001.

We next detected the local inflammatory level to determine whether SB supported the inhibition of the ligature-induced periodontal inflammation. As shown in Figure 3, the histomorphometric analyses of H&E staining images demonstrated that the sulcular epithelium and junctional epithelium in the P group were infiltrated with a large number of inflammatory cells showing characteristic morphology of large nuclei with dark blue-purple staining. Some of the inflammatory cells even infiltrated into the periodontal pocket. While the number of infiltrated inflammatory cells in the P+SB group was much less and limited in the epithelium without leaking into the periodontal pocket. The number of infiltrated inflammatory cells in the P group was significantly higher (7.6 times) than that in the C group (*P* < 0.0001) (Figures 3(a) and 3(b)). While the number of infiltrated inflammatory cells in the P+SB group (4.0 times in comparison with the C group) was significantly lower than that in the P group (*P* < 0.0001) (Figures 3(a) and 3(b)). The IHC staining of the periodontium showed that the intensities of NF-*κ*B and NLRP3 in the P group were significantly higher than those in the C group (*P* < 0.001) (Figure 3(c)). In contrast, the SB administration maintained the intensities of NF-*κ*B and NLRP3 at similar levels as those in the C group (*P* > 0.05) (Figures 3(c) and 3(d)). The mRNA expression levels of TNF*α*, IL-1*β*, and IL-6 in the P group were dramatically enhanced (8.2, 11.2, and 9.3 times) in comparison with those in the C group. The SB administration significantly attenuated such increases to much lower levels (3.1, 2.3, and 2.5 times respectively in comparison with the C group) (*P* < 0.0001) (Figure 3(e)).

### 3.4. SB Attenuated Periodontal OS

**Figure 4:**
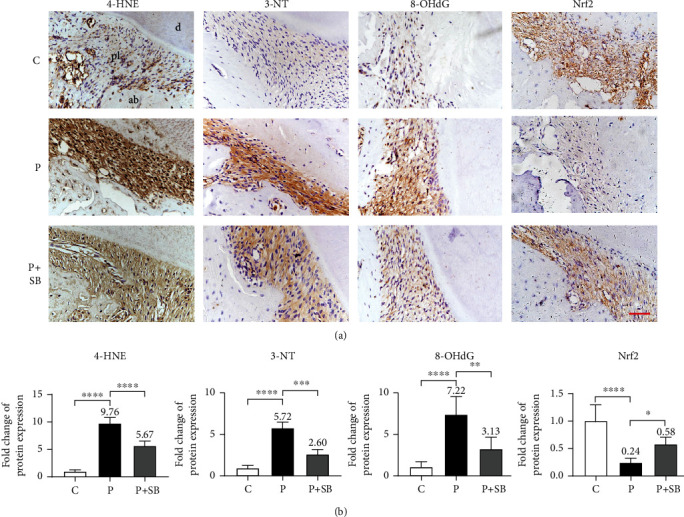
SB attenuated periodontal OS. (a) Immunohistochemistry staining images of 4-HNE, 3-NT, 8-OHdG, and Nrf2. pl: periodontal ligament; d: dentin; ab: alveolar bone. Scale bar = 50 *μ*m. (b) Protein expression intensity quantification analysis of 4-HNE, 3-NT, 8-OHdG, and Nrf2 in the periodontal ligament area (*n* = 5). The mean value of each group but group C was marked on the top of its column. Data are shown as mean ± SD. ^∗^*P* < 0.05, ^∗∗^*P* < 0.01, ^∗∗∗^*P* < 0.001, and ^∗∗∗∗^*P* < 0.0001.

In order to determine the OS level in the periodontal tissues, we further adopted IHC staining to assess the expression levels of 4-HNE, 3-NT, and 8-OHdG (Figure 4(a)). Quantitative analyses showed that the expression levels of 4-HNE, 3-NT, and 8-OHdG were substantially augmented (9.8, 5.7, and 7.2 times respectively) in the P group in comparison with those in the C group (*P* < 0.0001) (Figure 4(b)). Consistently, SB administration significantly attenuated such augmentation to much lower levels (5.7, 2.6, and 3.1 times respectively in comparison with the C group) (*P* < 0.001) (Figure 4(b)). In addition, we measured the expression level of Nrf2, the main transcription factor of antioxidant genes, using IHC staining. The intensity of Nrf2 in the P group was significantly lower (only 24.0% of the C group) than that in the C group, while the SB supplementation attenuated the reduction in Nrf2 protein level (about 57.5% of the C group), which was more than 2 times the level in the P group (*P* < 0.05) (Figures 4(a) and 4(b)).

### 3.5. SB Attenuated LPS-Induced Inflammation and OS in hPDLCs

**Figure 5:**
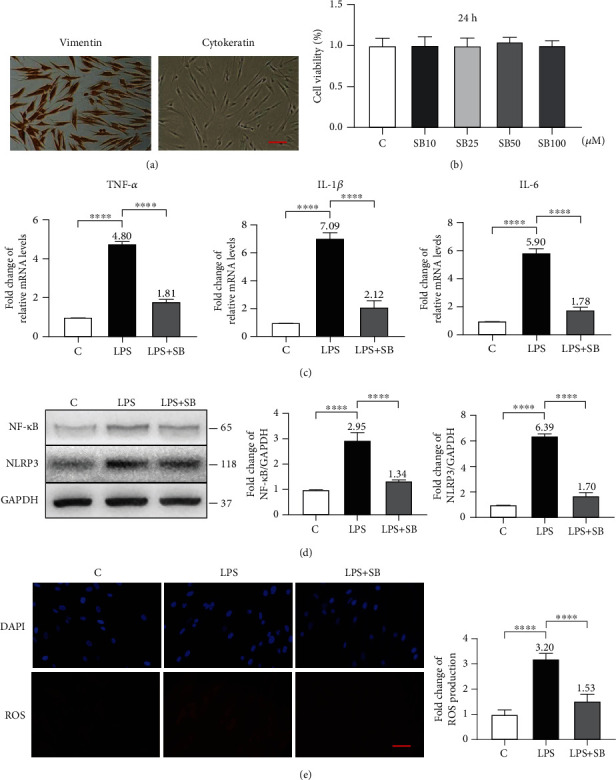
SB attenuated LPS-induced inflammation and OS in hPDLCs. (a) Immunohistochemical staining of hPDLCs. Positive staining for vimentin and negative staining for cytokeratin. Scale bar = 100 *μ*m. (b) hPDLCs were incubated with various concentrations (0–100 *μ*M) of SB for 24 h. Cell viability was assessed by MTT. (c) hPDLCs were exposed to 1 *μ*g/ml LPS with or without the preincubation of SB (50 *μ*M). mRNA expression of TNF-*α*, IL-1*β*, and IL-6 was measured by qPCR. GAPDH was used as a housekeeping gene. (d) Representative Western blotting bands and the quantification of NF-*κ*B and NLRP3 relative to GAPDH. (e) Representative DCFH-DA staining images and quantification of ROS production in indicated groups. Scale bar = 50 *μ*m. The mean value of each group but group C was marked on the top of its column. Data are shown as mean ± SD from three independent experiments. ^∗∗∗∗^*P* < 0.0001.

We characterized the spindle-shaped cells derived from PDL tissue using immunohistochemical staining and showed that they were positive for vimentin and negative for cytokeratin. This result indicates that these cells were typical hPDLCs (Figure 5(a)). The 24 h treatment with SB from 10 *μ*M to 100 *μ*M did not affect the viability of the hPDLCs (*P* > 0.05) (Figure 5(b)). After the incubation with 1 *μ*g/ml LPS for 24 h, the levels of proinflammatory cytokines, including TNF-*α*, IL-1*β*, and IL-6, in hPDLCs were significantly increased (4.8, 7.1, and 5.9 times respectively) in comparison with those in group C (*P* < 0.0001), whereas the pretreatment of 50 *μ*M SB for 24 h dramatically attenuated such increases to much lower levels (1.8, 2.1, and 1.8 times, respectively, in comparison with the C group) (*P* < 0.0001) (Figure 5(c)). Western blotting showed that in the LPS group, the expression levels of NF-*κ*B and NLRP3 were 2.9 and 6.4 times higher than those in the C group, respectively (*P* < 0.0001), whereas in the LPS+SB group, their levels (1.3 and 1.7 times in comparison with the C group) were significantly lower than those in the LPS group (*P* < 0.0001) (Figure 5(d)). The fluorescence intensity of DCFH-DA (as an indicator of intracellular ROS) was significantly enhanced (3.2 times) in the LPS group in comparison with that in the C group, while SB administration attenuated this enhancement to a much lower level (1.5 times in comparison with the C group) than that in the P group (*P* < 0.0001) (Figure 5(e)).

## 4. Discussion

Pharmaceutical reduction of inflammation and OS may present an efficacious approach to reduce periodontitis-induced periodontal tissue destructions so as to maximally preserve teeth. In this study, we assessed the efficacy of SB, a well-established anti-inflammatory and antioxidative compound, in reducing periodontitis-induced periodontal tissue destructions in rats. Our data show that SB administration maintained a similar ACJ-AC distance as that in the C group and substantially reduced the apoptosis of the PDLCs compared with the P group. We further show that the administration of SB was associated with dramatically lower levels of proinflammatory cytokines (TNF-*α*, IL-1*β*, and IL-6) and oxidative damage biomarkers (4-HNE, 3-NT, and 8-OHdG) in the rat model. Our *in vitro* data confirmed that SB could significantly suppress LPS-induced expression of the proinflammatory cytokines and intracellular ROS level in hPDLCs. These data suggested a promising application potential of SB in preventing periodontitis-induced periodontal tissue destructions.

In clinic, bacterial film always tends to accumulate on the surface of dental calculus, poor dental restorations, or dental overhangs, which contributes to the initiation of periodontitis. Considering the importance of periodontal pathogens, a possible method to establish animal models for periodontitis is to directly introduce exogenous bacteria into oral cavity through diet/drinking or by local microinjection [25]. However, such methods fail to mimic the process of bacteria accumulation in the oral cavity. As a promising alternative, placing ligature in the submarginal positions of molars provides a calculus/overhang-simulating complex for bacterial accumulation and plaque formation, leading to periodontal tissue destructions [26]. In our current study, we adopted the ligature-induced rat periodontitis model to investigate the effects of SB on preventing periodontal tissue destructions in periodontitis. We observed that after 2 weeks 35% alveolar bone resorption with typical horizontal alveolar bone loss and vertical bone defects was accompanied by connective tissue destruction, attachment loss, and collagen fiber breakdown comparing to that in the control group (Figures 2(a) and 2(b)), which indicated the successful establishment of the periodontitis model.

Natural compounds have been applied in the treatment of periodontitis to alleviate alveolar bone loss and improve clinical periodontal status in previous studies. The adjunctive use of natural compounds (e.g., curcumin and green tea extract) to scaling and root planing in chronic periodontitis patients significantly improves clinical outcomes, such as reduction of gingival index (GI), plaque index (PI), and probing depth (PD) [27, 28]. Resveratrol, with its anti-inflammatory and antioxidative properties, also suppresses periodontitis-mediated tissue damages in a rat model [29]. SB, the main active component of silymarin, has been shown to protect organs such as the liver, heart, and nervous system in many studies because of its anti-inflammatory and antioxidative properties [30, 31]. It has been well recognized that SB performs protective effects in inflammatory diseases such as rheumatoid arthritis [32], hepatitis [33], and pulmonary fibrosis [34]. SB also serves as a superb antioxidant retrieving redox balance [35] and bears a promising capacity of promoting bone regeneration [18]. Those functions make SB a promising agent to prevent the periodontitis-induced tissue destructions. In this study, after SB administration, we observed that about 24% of alveolar bone resorption was prevented in the P+SB group, the apoptosis of PDLCs also decreased more than 5-fold comparing to those in the P group (Figure 2), which ensures its protective effects in periodontitis prevention and therapy.

The process of periodontitis includes several inflammatory stages of tooth-supporting tissues [11]. The initial lesion stage is the response of resident leukocytes and endothelial cells to the bacterial biofilm, which induces the migration of neutrophils toward the site of inflammation. The early lesion stage follows by an increase in number of neutrophils in the connective tissue and the appearance of macrophages and T cells. As to the later established and advanced lesion stage, B cells and especially plasma B cells are dominant in the inflammation site [4, 36]. The infiltrated inflammatory cells release inflammatory mediators (e.g., proinflammatory cytokines such as TNF-*α*, IL-1*β*, IL-6, and chemokines) and tissue-degrading enzymes (e.g., collagenases and matrix metalloproteinases), which causes irreversible attachment loss and bone loss histologically and clinically [11]. In this study, H&E staining showed that a large number of inflammatory cells infiltrated in the periodontium of the rats in the P group, which was accompanied by alveolar bone resorption, collagen fiber breakdown and connective tissue destruction (Figures 2(b) and 3(a)). Our results showed that the presence of SB significantly inhibited inflammatory cell infiltration (Figures 3(a) and 3(b)) and prevented the increase of the relative mRNA expression of TNF-*α*, IL-1*β*, and IL-6 in both *in vivo* and *in vitro* models (Figures 3(e) and 5(c)), which indicates that SB effectively inhibited the inflammation in periodontitis.

One possible mechanism accounting for the inhibitory effect of SB on inflammation in periodontitis is by downregulation of NF-*κ*B, a pivotal transcriptional regulator of proinflammatory cytokines and chronic inflammation [37]. In the progression of periodontitis in human, NF-*κ*B can be activated by LPS-induced Toll-like receptor 4 (TLR4) activation, which activates gene expression of multiple proinflammatory cytokines, such as IL-1, IL-6, and TNF-*α* [7]. NF-*κ*B also enhances the expression of NLRP3 and potentiates the activity of NLRP3 inflammasome [38], which triggers the maturation and secretion of IL-1*β* [39]. Previous studies have shown that expression levels of NF-*κ*B and NLRP3 are significantly elevated in the periodontitis animal models, while chemical inhibitors of NF-*κ*B and NLRP3 or their downregulation can significantly reduce the periodontitis-induced alveolar bone loss [40–43]. In line with these results, in the present study, the expression levels of NF-*κ*B and NLRP3 in the P group were significantly higher than those in the control group. We further showed that SB administration significantly attenuated the expression levels of NF-*κ*B and NLRP3 in the P+SB group in comparison with those in the P group (Figures 3(c), 3(d), and 5(d)). Consistently, SB also significantly downregulated the expression levels of proinflammatory cytokines (TNF-*α*, IL-1*β*, and IL-6). Therefore, the downregulation of the NF-*κ*B/NLRP3 axis might be involved in the anti-inflammatory effects of SB in periodontitis.

In addition to inflammation, ROS also contributes to the progression of periodontitis. ROS is mainly produced by inflammatory cells to manage cell signaling, gene regulation, and antimicrobial defense [12]. In the pathogenesis of periodontitis, continuous pathogenic stimuli stimulate excessive release of ROS, which results in an imbalance in redox homeostasis, finally leading to OS. In the OS status, excessive ROS attacks and causes dysfunction to mitochondria of periodontal cells, whose damages further stimulate production of ROS, thereby exacerbating OS and oxidative damages to periodontal tissues [44]. The oxidative damage includes lipid peroxidation, protein denaturation, and DNA damage, which cause dysfunction and apoptosis of periodontal cells, further contributing to periodontal destructions [45]. Our previous report has shown that the changes of 4-HNE, 3-NT, and 8-OHdG—indicators of lipid, protein, and DNA oxidative damages, respectively—have a positive correlation with periodontal destructions [20]. In our *in vitro* model, LPS increased the production of intracellular ROS in hPDLCs as shown by the enhanced fluorescence intensity of DCFH-DA (Figure 5(e)). In our *current in vivo* model, the expression levels of 4-HNE, 3-NT, and 8-OHdG were increased in the P group (Figures 4(a) and 4(b)). In contrast, SB administration significantly attenuated the production of intracellular ROS in LPS-stimulated hPDLCs (Figure 5(e)), and the expression levels of 4-HNE, 3-NT, and 8-OHdG in the experimental periodontitis rat model were also significantly reduced (Figures 4(a) and 4(b)). As a polyphenolic flavonoid, SB has a strong innate free radical scavenging activity [46]. It can react directly with free radicals and forms a more stable and harmless flavonoid radical [19]. Meanwhile, SB also can increase the activity of antioxidant enzymes, such as catalase (CAT), superoxidase dismutase (SOD), glutathione peroxidase (GPX), and heme oxygenase 1 (HO-1) [47, 48], so as to eliminate free radicals.

Gene expression of a series of antioxidant enzymes is regulated by Nrf2, a redox-sensitive transcription factor. Under unstressed conditions, Nrf2 remains in the cytoplasm and is repressed by binding to Kelch-like ECH-associating protein 1 (Keap1), a negative regulator that mediates the subsequent degradation of Nrf2 by proteasome [49]. When exposed to oxidative stimulus, Nrf2 is unhinged from Keap1, translocates into the nucleus, and binds to the antioxidant response element (ARE), inducing the transcription of antioxidant enzymes including GPX, glutathione S-transferase (GST), and HO-1. These Nrf2/ARE-driven effectors further eliminate multiple ROS, exerting their superb antioxidative capacity [50]. It is well established that Nrf2 is a very important target for polyphenols like SB to attain their antioxidative activities [51]. SB is demonstrated to activate the Nrf2 signaling pathway in many disease models [51, 52]. However, whether SB could activate the Nrf2 signaling pathway and attenuate the oxidative damages in periodontitis has yet not been reported. Therefore, in the present study, we detected the expression of Nrf2 and observed that SB largely retained Nrf2 levels in group P+SB (Figures 4(a) and 4(b)), which might account for the rehabilitation of disordered redox status of periodontitis.

In the current study, we show that SB significantly attenuated periodontal tissue damages in experimental periodontitis via downregulating inflammation and OS. Further studies about the underlying molecular mechanism and large animal models as well as clinic trials are needed in the future to confirm the protective effects of SB in periodontitis.

## 5. Conclusions

SB administration can downregulate periodontitis-induced inflammation and OS, so as to attenuate periodontal tissue destruction in experimental periodontitis. Our findings suggest an interesting prospect in the potential clinical application of SB in periodontitis prevention and therapy.

## Figures and Tables

**Table 1:** Primers sequences for polymerase chain reaction (PCR).

Gene (rat)	Forward/reverse	Sequence
Interleukin-1*β* (IL-1*β*)	F:	5′-GCTGTCCAGATGAGAGCATC-3′
	R:	5′-GTCAGACAGCACGAGGCATT-3′

Interleukin-6 (IL-6)	F:	5′-AGACTTCCAGCCAGTTGCCT-3′
	R:	5′-CTGACAGTGCATCATCGCTG-3′

Tumor necrosis factor-*α* (TNF-*α*)	F:	5′-AGGACACCATGAGCACGGAA-3′
	R:	5′-GGGCCATGGAACTGATGAGA-3′

Glyceraldehyde-3-phosphate dehydrogenase (GAPDH)	F:	5′-TCTCTGCTCCTCCCTGTTCT-3′
	R:	5′-CTTGCCGTGGGTAGAGTCAT-3′

**Table 2:** Primers sequences for polymerase chain reaction (PCR).

Gene (human)	Forward/reverse	Sequence
Interleukin-1*β* (IL-1*β*)	F:	5′-GGACAAGCTGAGGAAGATGC-3′
	R:	5′-TCGTTATCCCATGTGTCGAA-3′

Interleukin-6 (IL-6)	F:	5′-CACAGACAGCCACTCACCTC-3′
	R:	5′-TTTTCTGCCAGTGCCTCTTT-3′

Tumor necrosis factor-*α* (TNF-*α*)	F:	5′-AGCCCATGTTGTAGCAAACC-3′
	R:	5′-TGAGGTACAGGCCCTCTGAT-3′

Glyceraldehyde-3-phosphate dehydrogenase (GAPDH)	F:	5′-CGAGATCCCTCCAAAATCAA-3′
	R:	5′-TTCACACCCATGACGAACAT-3′

## Data Availability

The data used to support the findings of this study are available from the corresponding author upon request.
